# Effect of body mass index on 30-day complication rate and implant survival rate after simultaneous bilateral unicompartmental knee arthroplasty: a multicentre retrospective study

**DOI:** 10.1186/s12891-024-07639-z

**Published:** 2024-07-05

**Authors:** Kuishuai Xu, Tianrui Wang, Tengbo Yu, Xia Zhao, Yingze Zhang, Liang Zhang

**Affiliations:** 1 Department of Sports Medicine, the Affiliated Hospital of Qingdao University, Shandong Qingdao, 266000 China; 2 Department of Traumatology, the Affiliated Hospital of Qingdao University, Shandong Qingdao, 266000 China; 3 Department of Orthopedic Surgery, University of Health and Rehabilitation Sciences (Qingdao Municipal Hospital), Shandong Qingdao, 266000 China; 4 Department of Abdominal ultrasound, the Affiliated Hospital of Qingdao University, Shandong Qingdao, 266000 China

**Keywords:** Unicompartmental knee arthroplasty, Knee, Body mass index, Obesity

## Abstract

**Objective:**

The practice of simultaneous bilateral unicompartmental knee arthroplasty (SBUKA) remains a topic of debate, particularly in patients with obesity. Thus, the purpose of this study was to assess the impact of body mass index (BMI) on the 30-day complication rate and the survival rate of the implant following SBUKA.

**Methods:**

We retrospectively examined the clinical records of 245 patients (490 knees) who underwent SBUKA at the Affiliated Hospital of Qingdao University and the Third Hospital of Hebei Medical University between January 2010 and December 2020. Patients were categorised based on their BMI at the time of surgery into four groups: normal weight (BMI 18.5 to 22.9 kg/m^2^), overweight (BMI 23.0 to 24.9 kg/m^2^), obese (BMI 25.0 to 29.9 kg/m^2^), and severely obese (BMI ≥30 kg/m^2^). Variables such as length of hospital stay, duration of surgery, and costs of hospitalisation were compared across all groups. Additionally, we recorded the 30-day postoperative complication rate and the time from surgery to any required revision. The Kaplan-Meier survival analysis was employed to evaluate and compare the implant survival rates.

**Results:**

The follow-up period for the 245 patients ranged from 39 to 114 months, with an average of 77.05±18.71 months. The incidence of complications within 30 days post-surgery did not significantly differ across the groups (χ2 = 1.102, *p* = 0.777). The implant survival rates from the lowest to the highest BMI groups were 97.14%, 93.9%, 94.44%, and 96.43%, respectively. Both the rate of implant revision (χ2 =1.612, *p* = 0.657) and the survival curves of the implants (*p* = 0.639) showed no statistically significant differences among the groups.

**Conclusions:**

BMI did not influence the 30-day complication rate nor the survival rate of implants following SBUKA, suggesting that SBUKA should not be contraindicated based on BMI alone.

## Introduction

Knee osteoarthritis (KOA) represents a prevalent chronic degenerative condition of the knee joint, significantly impacting the quality of life among middle-aged and elderly populations [1]. The pathological progression of KOA predominantly affects the medial joint space in its early to middle stages, due to the lower limb's force line and the knee joint's anatomical characteristics. Artificial unicondylar knee arthroplasty (UKA) has been demonstrated to offer substantial therapeutic benefits in these cases [2]. Statistical evidence indicates that one-third of individuals with KOA experience bilateral affliction, with approximately 10%-20% undergoing knee arthroplasty on the contralateral knee due to symptom exacerbation in the opposite joint [3, 4]. This subsequent intervention, commonly termed staged bilateral UKA, is associated with an elevated risk of perioperative complications, attributable to repeated hospital admissions and the cumulative effects of anaesthesia [5]. Consequently, simultaneous bilateral UKA (SBUKA) presents an appealing alternative to mitigate the challenges posed by staged surgeries. Nonetheless, the impact of SBUKA on postoperative complication rates and implant longevity remains a subject of debate. Prior research has reported a marked increase in complication rates following SBUKA, including elevated incidences of blood transfusion, mortality, pulmonary embolism, and deep vein thrombosis, compared to unilateral or staged bilateral UKA [6, 7].

Furthermore, an increasing body of evidence suggests that body mass index (BMI) may influence the likelihood of prosthesis revision following UKA [8–11]. Despite UKA's reported ten-year survival rate exceeding 90%, its revision frequency is higher in comparison to total knee arthroplasty (TKA) [12]. To reduce revision rates and other adverse outcomes, stringent selection criteria for UKA candidates have been advocated, incorporating considerations for obesity [13]. However, the effect of elevated BMI on implant durability continues to be contentious. Previous research has established morbid obesity as an independent risk factor for compromised functional outcomes and reduced implant longevity post-UKA [8, 9]. A meta-analysis highlighted that obese patients face an increased risk of revision, including aseptic revision and other complications following UKA [10]. The incidence of early prosthesis revision in morbidly obese patients is more than quintuple that of other patients, predominantly due to disease progression in adjacent compartments or instability of the mobile bearing [11].

Current studies have focused more on the effect of BMI on postoperative complications and prosthesis revision after unilateral UKA, but in the absence of direct comparisons of complications and revision rates between specific obesity classes in SBUKA, the effect of obesity remains uncertain. At the same time, it remains unknown whether SBUKA can be one of the best treatments for obese patients with bilateral knee osteoarthritis. In order to overcome the limitations of small sample size, small number of revision cases, and short follow-up time, a multi-center retrospective study was conducted to investigate the effect of BMI on the 30-day post-SBUKA complication rate and prosthesis revision rate.

## Materials and methods

### Inclusion and exclusion criteria

The clinical records of 245 patients (490 knees) who underwent simultaneous bilateral unicompartmental knee arthroplasty (SBUKA) at the Affiliated Hospital of Qingdao University and the Third Hospital of Hebei Medical University from January 2010 to December 2020 were retrospectively reviewed. Inclusion criteria included: (1) Patients aged over 18 and under 85 years with indications for UKA as per Goodfellow’s recommendation and the Oxford Radiological Decision Aid; (2) Presence of anteromedial osteoarthritis in both knee joints; (3) Normal functioning of the medial collateral and anterior cruciate ligaments in both knees; (4) Less than 15° of flexion contracture; (5) Acceptable patellofemoral joint condition; (6) Bilateral knee osteoarthritis necessitating SBUKA. Exclusion criteria encompassed: (1) Previous knee surgery (meniscectomy, ligament reconstruction, high tibial osteotomy, arthroplasty, tibial plateau fractures); (2) BMI < 18.5 kg/m^2^; (3) Patients lost to follow-up; (4) Concurrent surgeries alongside SBUKA.

The following formula was used to determine the patient's BMI [14]. The study groups were classified according to the BMI classification standard for Asian adults as defined by the World Health Organization (WHO) [15]. According to the BMI at the time of surgery, the patients were divided into normal group (*n*=35), overweight group (*n*=41), obesity group (*n*=99), and severe obesity group (*n*=70). The study was approved by the Ethics committee of the Affiliated Hospital of Qingdao University(Approval No.: QYFYWZLL28296), and all patients signed informed consent forms.

### Surgical procedures

Patients were administered general anaesthesia and positioned supine. A pneumatic tourniquet was applied at the thigh's proximal end following exsanguination. A medial parapatellar approach was employed, with incisions ranging from 6 to 8 cm through the skin, subcutaneous tissue, and joint capsule, sequentially. This facilitated exposure of the medial tibiofemoral compartment, where the integrity of the anterior cruciate ligament, medial collateral ligament, and lateral tibiofemoral compartment was verified. Osteotomies were performed using an extramedullary guide for the tibia and a specialised femoral guide for the distal end of the femoral internal condyle. The positioning of the femoral prosthesis was determined, followed by the fitting and sizing of the prosthesis model. Adjustments to the prosthesis size were made as necessary. The joint cavity was lavaged with saline, the prosthesis dried, and the selected monocondylar prosthesis implanted using cryo-cured bone cement. Following cement curing, excess cement was removed, and knee joint mobility and prosthesis stability were assessed through flexion and extension movements. The tourniquet was released, achieving complete haemostasis of the incision and joint cavity, a negative pressure drainage tube was placed, and the incision sutured. Finally, an elastic bandage was applied under pressure.

### Outcomes

Data on surgical age, gender, weight, height, follow-up duration, hospital stay length, operative time, and hospitalisation costs were collected for all patients, with complications monitored 30 days post-surgery. The incidence and reasons for prosthesis revision at the final follow-up were analysed.

### Statistical analysis

SPSS23.0 statistical software was used for analysis. The measurement data were expressed as mean±standard deviation, and one-way analysis of variance (ANOVA) was used for comparison between groups. Counting data were expressed as cases and percentages, and Chi-square test was used for comparison between groups. The renovations were analyzed using Kaplan-Meier survival curves. Significance was set at *P* < 0.05.

## Results

### Comparison of baseline data in four groups

**Fig. 1:**
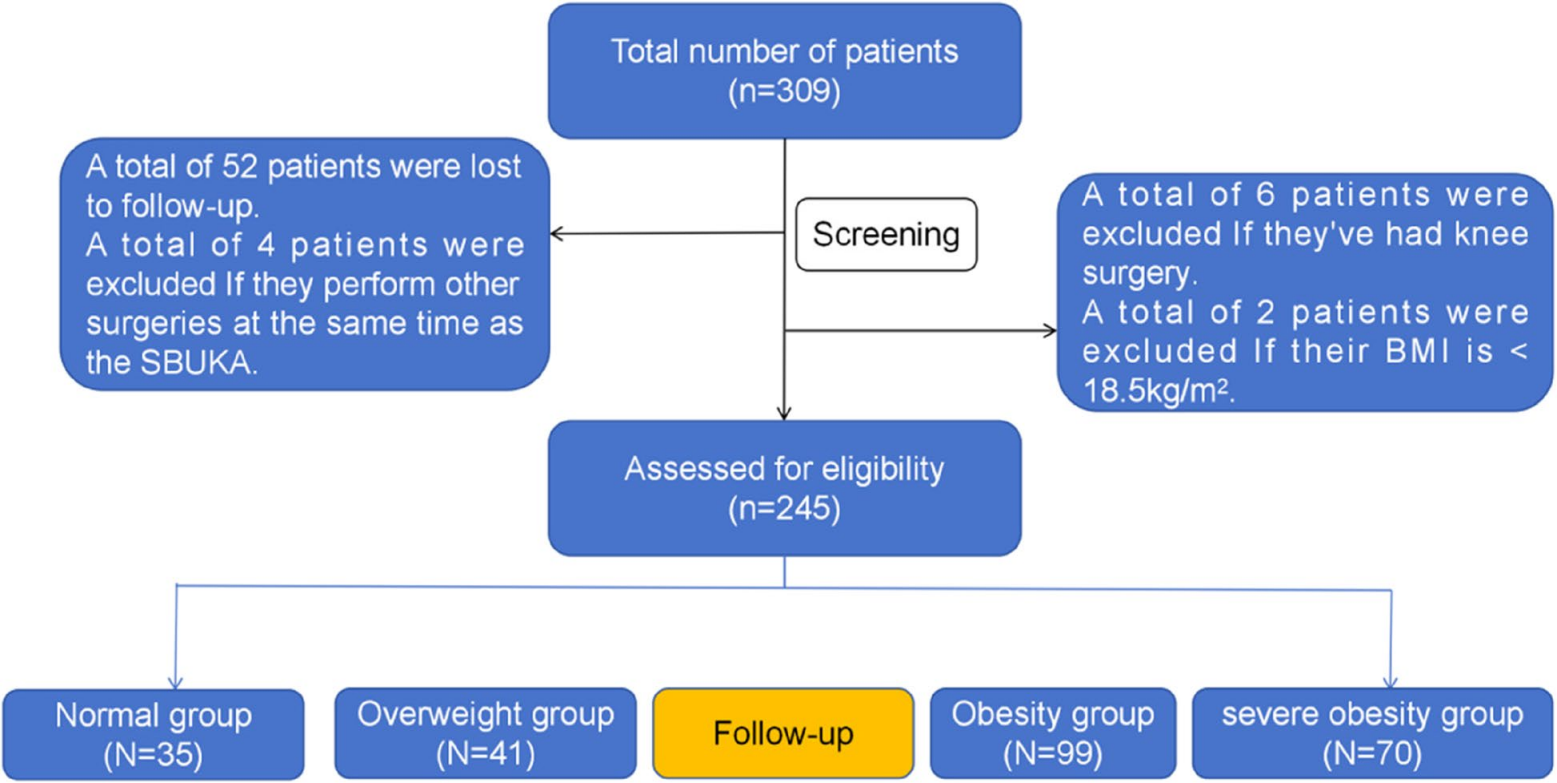
Flowchart showing the retrospectively identifed cohort of patients who underwent SBUKA at our study and the reasons for exclusion

Flowchart showing the retrospectively identifed cohort of patients who underwent SBUKA at our study and the reasons for exclusion (Fig. 1). A total of 52 patients were lost to follow up. Therefore, their revision status remained unknown. In the end, 245 patients were enrolled in the study. According to the BMI at the time of surgery, the patients were divided into normal group (*n*=35), overweight group (*n*=41), obesity group (*n*=99), and severe obesity group (*n*=70). The 245 patients were followed up for 39 to 114 months, with an average of (77.05±18.71) months. There were 57 men and 188 women. Age, gender, follow-up time, hospital stay, operation time and hospitalization cost were compared among the four groups, and there was no statistical significance (*p* > 0.05) (Table 1).

**Table 1:** Comparison of baseline data in four groups

Variables	Normal group (*N*=35)	Overweight group (*N*=41)	Obesity group (*N*=99)	severe obesity group (*N*=70)	F/χ2	*p* value
Sex					0.482	0.923^a^
Male, n	9	8	23	17		
Female, n	26	33	76	53		
Age (years, mean ± SD)	62.66±6.69	61.44±6.99	60.75±7.51	58.93±7.66	2.307	0.077^b^
Mean follow-up(months, mean ± SD)	73.4±19.59	76.29±19.57	75.97±19.24	80.84±16.66	1.545	0.203^b^
Hospital stay (days, mean ± SD)	8.63±3.46	7.78±1.71	8.55±3.47	9.19±2.66	1.901	0.13^b^
Hospitalization costs ($, mean ± SD)	9794.03±3555.37	9436.12±2286.9	9636.15±2717.76	103377.03±3174.28	1.097	0.351^b^
Time of operation (mins, mean ± SD)	120.34±22.39	122.68±39.29	128.82±30.19	127.63±30.45	0.875	0.454^b^

*N* Number of patients, *SD* Standard deviation

^a^Pearson chi-Square test

^b^ANOVA test

### Complications 30 days after SBUKA

The distribution of complications and outcomes across the BMI categories is presented in Table 2. The Normal group experienced four complications, comprising two instances of transfusion, one readmission, and one superficial surgical site infection (SSI). In the Overweight group, there were three complications: one blood transfusion, one urinary tract infection, and one superficial SSI. The Obesity group reported six complications, including three blood transfusions, one urinary tract infection, one reoperation, and one case of deep vein thrombosis. The Severe Obesity group had five complications, with one blood transfusion, one case of pneumonia, two cases of superficial SSI, and one case of deep vein thrombosis. No significant difference was observed in the incidence of complications 30 days post-surgery across all groups (χ^2^ = 1.102, *p* = 0.777).

**Table 2:** 30-day complication rates in four groups

Outcome	Normal group (*N*=35)	Overweight group (*N*=41)	Obesity group (*N*=99)	Severe obesity group (*N*=70)	χ2	*p* value
Superficial SSI	1	1	0	2		
Pneumonia	0	0	0	1		
Urinary tract infection	0	1	1	0		
Transfusions within 72 h of surgery	2	1	3	1		
Deep vein thrombosis	0	0	1	1		
Reoperation	0	0	1	0		
Readmission	1	0	0	0		
Any complication	4	3	6	5	1.102	0.777

*N* Number of patients, *SSI* Surgical site infection

### Implant revision status

Within the scope of this study, 23 knees required implant revision (Table 3). Ten knees underwent total knee arthroplasty (TKA) due to the postoperative progression of lateral compartment osteoarthritis. Nine knees experienced dislocation of the polyethylene insert for various reasons. Aseptic loosening was noted in two knees. One knee joint developed a periprosthetic joint infection, and one knee exhibited unexplained liner wear. Revision incidences were as follows: two in the Normal group, five in the Overweight group, eleven in the Obesity group, and five in the Severe Obesity group. The comparison of complications occurring within 30 days post-SBUKA across the four groups revealed no statistically significant difference (χ^2^ =1.612, *p*=0.657) (Table 4).

**Table 3:** Reason for implant revision in four groups

Normal group (*N*=35)		Overweight group (*N*=41)		Obesity group (*N*=99)		Severe obesity group (*N*=70)	
Reason for revision	Rate	Reason for revision	Rate	Reason for revision	Rate	Reason for revision	Rate
Lateral interventricular osteoarthritis	1/70	Lateral interventricular osteoarthritis	2/82	Lateral interventricular osteoarthritis	5/198	Lateral interventricular osteoarthritis	2/140
Periprosthetic joint infection	-	Periprosthetic joint infection	-	Periprosthetic joint infection	1/198	Periprosthetic joint infection	-
Aseptic loosening	-	Aseptic loosening	1/82	Aseptic loosening	1/99	Aseptic loosening	-
Gasket dislocation	1/70	Gasket dislocation	2/82	Gasket dislocation	3/198	Gasket dislocation	3/140
Liner wear	-	Liner wear	-	Liner wear	1/198	Liner wear	-
Total	2/70	Total	2/82	Total	11/198	Total	5/140

**Table 4:** Survival rate of the implant in four groups

BMI group	N knees	Number of revision	Survival rate	χ2	p value
Normal group (*N*=35)	70	2	97.14%		
Overweight group (*N*=41)	82	5	93.9%		
Obesity group (*N*=99)	198	11	94.44%		
Severe obesity group (*N*=70)	140	5	96.43%		
Entire cohort	490	23	95.31%	1.612	0.657

*N* = number of patients

### Implant survival rate

**Fig. 2:**
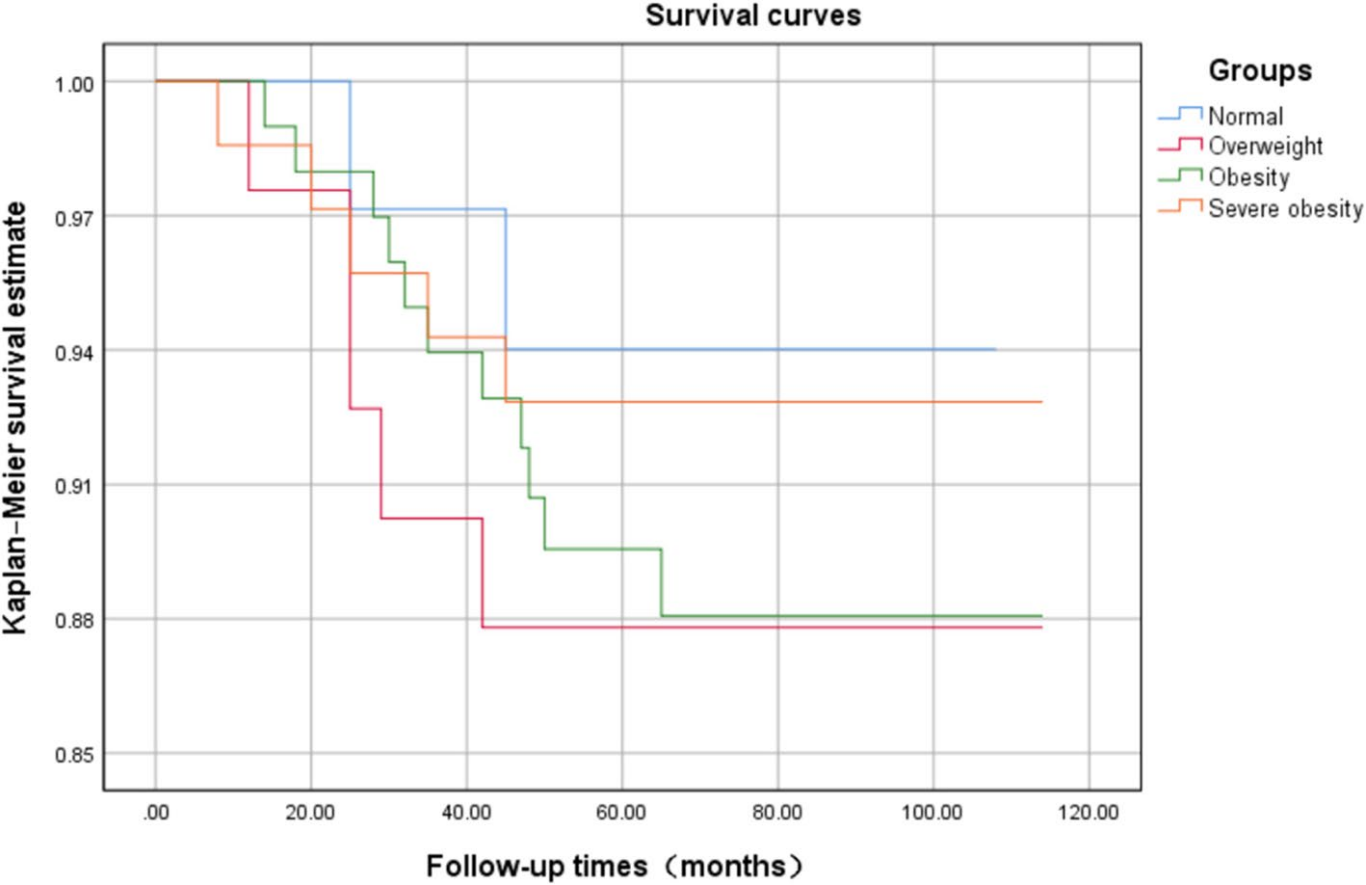
The revision was evaluated using Kaplan-MeierKaplan-Meier survival analysis. No difference in the prosthesis survival curve was found among the four groups (*p* = 0.639)

From the lowest BMI to the highest, the implant survival rate were 97.14%, 93.9%, 94.44% and 96.43%. It can be observed from the Kaplan-Meier prosthetic survival curve of the four groups that the prosthetic retention rates of the four groups gradually decreased with the increase of follow-up time until the last follow-up (Fig. 2). No difference in the prosthesis survival curve was found (*p* = 0.639).

## Discussion

Previous investigations have assessed the perioperative complications and necessity for prosthesis revision in cases of simultaneous bilateral unicompartmental knee arthroplasty (SBUKA) [6, 7]. However, the safety of this procedure in obese patients has yet to be definitively established. Notably, extant research has predominantly concentrated on patients undergoing unilateral UKA [8–11], and it is uncertain whether these findings are directly transferable to individuals receiving SBUKA. Given the absence of a significant disparity in both the 30-day complication rate and the prosthesis revision rate post-SBUKA across varying BMI categories, the present outcomes do not corroborate these earlier reports [6–11]. These findings imply that BMI should not automatically preclude candidates with bilateral osteoarthritis from considering SBUKA.

The prevalence of obesity among patients undergoing UKA is increasingly documented. Large-scale studies from both the United States and the United Kingdom have indicated that the mean BMI for UKA patients was reported as 29.24 [16] and 29.9 kg/m^2^ [17], respectively. Although obesity is traditionally viewed as a contraindication for UKA, the criteria for selecting UKA candidates remain a matter of debate and exhibit significant variability. Complicating this issue further, there exists a plethora of contradictory evidence regarding the impact of obesity on the risk of both short-term and long-term complications following unilateral primary UKA [18–21], with a notable absence of research concerning the effects of obesity on outcomes post-SBUKA. Several studies have identified a correlation between obesity and complications in UKA recipients. For instance, a higher BMI has been associated with a reduced level of postoperative activity [18], identified as an independent risk factor for delayed wound healing post-UKA [19], and linked to an increased probability of 90-day readmission [20]. Nonetheless, a meta-analysis has suggested that obesity does not necessarily elevate complication or infection rates following UKA, arguing against the classification of obesity as a contraindication for the procedure [21]. To our knowledge, this study is the first to examine the incidence of complications post-SBUKA across different BMI ranges. Our analysis reveals no significant difference in the 30-day complication rate post-SBUKA among various BMI groups. Although the duration of surgery was longer in obese patients compared to their normal-weight counterparts, this variance did not reach statistical significance.

We conducted a meticulous analysis of the occurrence of complications within each group. Among all noted complications (18 cases), blood transfusions were most prevalent (7 cases, 38.89%) within the first 72 hours post-surgery. Previous literature corroborates that the rate of blood transfusion in patients undergoing SBUKA is significantly elevated compared to those receiving unilateral UKA [6]. This increased requirement may be attributed to the longer duration and more extensive trauma associated with SBUKA, which can lead to a greater volume of intraoperative blood loss. Incisional complications remain a common issue in orthopaedic surgery; in this study, SSIs were observed in four knees (22.22%), though there were no instances necessitating revision due to periprosthetic infection. Notably, surgical wound healing is impeded in patients with obesity. A BMI greater than 32.0 kg/m^2^ has been identified as an independent risk factor for delayed wound healing post-UKA [20], primarily due to the excessive subcutaneous fat and a relatively unchanged vascular network, which can adversely affect healing. Moreover, obese patients face a heightened risk of incisional fat liquefaction, potentially leading to infection post-SBUKA.

In addition to influencing short-term complications, obesity critically affects the long-term survival of UKA implants. This study documented 23 cases of implant revision, with 6 knees requiring TKA due to postoperative progression of lateral compartment osteoarthritis and 7 knees undergoing TKA for loosening of femoral or tibial components. Dislocation of the polyethylene insert, attributed to various causes, was observed in 10 knees. Contrasting with a meta-analysis of 3,967 UKA revisions [9]—where aseptic loosening was the primary cause of revision, accounting for approximately 36%—our findings highlight polyethylene insert dislocation as a predominant reason for prosthesis revision in SBUKA patients. This discrepancy could be due to the majority of prostheses in this study being of the mobile-bearing type, with dislocation often occurring early postoperatively. Our findings significantly exceed the rates reported in prior literature (2% to 4%) [22]. Bae et al. [23] noted that dislocation was largely due to surgical technicalities such as improper prosthesis alignment and joint space imbalance, more commonly observed in Asian populations, possibly related to frequent squatting, kneeling, and similar postures in daily activities. Additionally, a posterior tibial slope greater than 7 degrees postoperatively was identified to enlarge the flexion gap excessively, thereby elevating the risk of insert dislocation. It is advisable to minimize excessive posterior tibial slope [24]. Aseptic loosening remains a significant cause for prosthesis revision post-SBUKA, with studies by Saragaglia [25] and Barrett et al. [26] indicating that isolated tibial lateral loosening was most common, followed by bilateral loosening, and isolated femoral lateral loosening being least frequent. Given the limited sample size, revisions were few across the groups. Future research should aim to enlarge the sample size and more comprehensively compare the reasons for prosthesis revision among the different groups.

Numerous studies have deduced that obesity does not negatively impact the survival or revision rates following UKA. Historically, obesity was deemed a contraindication for fixed-bearing UKA owing to concerns about accelerated polyethylene wear and implant loosening [27]. However, contemporary research indicates that BMI does not compromise implant survival post-UKA [28]. A meta-analysis further substantiated that obesity does not elevate the risk of prosthesis revision [21]. To date, there have been no medium- to long-term investigations examining the influence of BMI on prosthetic retention following SBUKA. This study revealed no significant variance in prosthetic retention rates across different BMI categories, nor did it observe a declining trend in prosthetic retention with increasing BMI. These findings align with prior research on unilateral UKA. Our conclusions are corroborated by Murray et al. [29], whose research demonstrated that prosthetic longevity in 2,438 UKA patients was not adversely affected by higher BMI. Similar outcomes were reported by Kuipers et al. [30] after an average follow-up period of 2.6 years. Additionally, Naal et al. [31] and Xing et al. [32] found that prosthesis survival rates were comparable between non-obese and obese patients undergoing UKA, after mean follow-up durations of 2 years and 4.5 years, respectively.

Our study has several limitations. First, because this was a multi-center retrospective study, there were differences in surgical techniques and proficiency among different surgeons. However, the amount of surgery that can be achieved by the same operator cannot meet the sample size required for experimental studies. Secondly, this study still has the problem of insufficient sample size, especially the patients with BMI>40kg/m^2^ or BMI>35kg/m^2^ were few, so we did not conduct more detailed grouping. Future studies should focus on people with higher BMI to more accurately reflect the risk of obesity. Finally, this study is a retrospective study, with the possibility of selection bias. The follow-up time is short, and longer studies are needed to clarify the long-term clinical outcomes of this technique.

## Conclusions

BMI did not influence the 30-day complication rate nor the survival rate of implants following SBUKA, suggesting that SBUKA should not be contraindicated based on BMI alone.

## Data Availability

The datasets generated during and/or analysed during the current study are available from the corresponding author on reasonable request.

## Abbreviations

KOA: Knee osteoarthritis
UKA: Unicompartmental knee arthroplasty
TKA: Total knee arthroplasty
BMI: Body mass index
ANOVA: Analysis of Variance
SBTKA: Simultaneous bilateral unicompartmental knee arthroplasty
WHO: World Health Organization
